# Green highly sensitive and selective spectroscopic detection of guaifenesin in multiple dosage forms and spiked human plasma

**DOI:** 10.1038/s41598-024-68711-1

**Published:** 2024-08-12

**Authors:** Neven N. Mikawy, Nancy Magdy, Marwa H. Mohamed, Amira M. El-Kosasy

**Affiliations:** Department of Pharmaceutical Analytical Chemistry, Faculty of Pharmacy Pharmaceutical Analytical Chemistry Department, Faculty of Pharmacy, Ain Shams University, Abbassia, 11566 Cairo Egypt

**Keywords:** Guaifenesin, Spectrofluorimetry, Chemometry, Green metric tools, Spiked human plasma, Biochemistry, Medical research

## Abstract

Guaifenesin (GUA) is determined in dosage forms and plasma using two methods. The spectrofluorimetric technique relies on the measurement of native fluorescence intensity at 302 nm upon excitation wavelength “223 nm”. The method was validated according to ICH and FDA guidelines. A concentration range of 0.1–1.1 μg/mL was used, with limit of detection (LOD) and quantification (LOQ) values 0.03 and 0.08 µg/mL, respectively. This method was used to measure GUA in tablets and plasma, with %recovery of 100.44% ± 0.037 and 101.03% ± 0.751. Furthermore, multivariate chemometric-assisted spectrophotometric methods are used for the determination of GUA, paracetamol (PARA), oxomemazine (OXO), and sodium benzoate (SB) in their lab mixtures. The concentration ranges of 2.0–10.0, 4.0–16.0, 2.0–10.0, and 3.0–10.0 µg/mL for OXO, GUA, PARA, and SB; respectively, were used. LOD and LOQ were 0.33, 0.68, 0.28, and 0.29 µg/mL, and 1.00, 2.06, 0.84, and 0.87 µg/mL for PARA, GUA, OXO, and SB. For the suppository application, the partial least square (PLS) model was used with %recovery 98.49% ± 0.5, 98.51% ± 0.64, 100.21% ± 0.36 & 98.13% ± 0.51, although the multivariate curve resolution alternating least-squares (MCR-ALS) model was used with %recovery 101.39** ± **0.45, 99.19** ± **0.2, 100.24 ± 0.12, and 98.61 ± 0.32 for OXO, GUA, PARA, and SB. Analytical Eco-scale and Analytical Greenness Assessment were used to assess the greenness level of our techniques.

## Introduction

**Figure 1:**
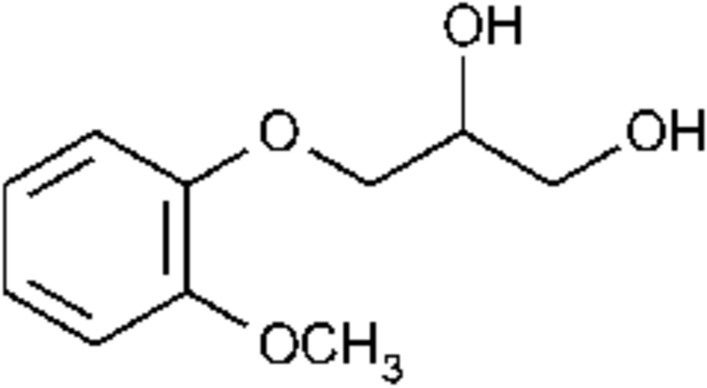
Chemical structure of guaifenesin.

Guaifenesin (GUA) is offered as a cough and cold remedy. GUA is chemically 3-(2-methoxyphenoxy) propane-1,2-diol^1^ (Fig. 1). GUA medications are used to treat the breathing-related symptoms of colds, allergies, asthma, bronchitis, sinusitis, and other illnesses (coughing, runny/stuffy nose, congestion)^2^. Since it is believed to lessen cough discomfort by increasing sputum volume and decreasing sputum viscosity, which in turn facilitates effective coughing, the most common GUA application for coughing is known as an expectorant^3^. Guaifenesin's indication is listed in the Food and Drug Administration's (FDA) Over-the-Counter (OTC) Monograph as "helping loosen mucus and thin bronchial secretions in patients with stable chronic bronchitis," but there is little information available regarding the medication's action or clinical efficacy in this condition^4^.

Fluorescence is one of the spectroscopic techniques that are first utilized in pharmaceutical examination. Fluorimetry has recently improved in terms of instrumentation and sample processing, making it far more sensitive, selective, and applicable to regular analysis^5^. Additionally, fluorescence is an adaptable analytical technique that is more sensitive than other detection methods like conventional UV absorption, less expensive than LC–MS detection, and faster than HPLC^6–9^.

Chemometrics is a branch of chemistry that designs or chooses the best measurement processes and experiments using formal logic. It also employs chemical data analysis to deliver the most relevant chemical information possible^10^. Chemometric approaches are frequently employed when many wavelengths that are tailored to the target molecule cannot be found. If there are two components that contribute to the spectrum, Beer's law can be expanded to account for the distinct contributions of each component^11^.Although chemometrics uses a variety of tools, principal components analysis (PCA), partial least squares (PLS), multivariate curve resolution (MCR) alternating least-squares (ALS) are the most popular^12^.

Different instrumental methods have been reported for the determination of GUA, involving chromatographic techniques^13–29^, electrochemical techniques^30–38^, and spectroscopic method^39–45^.

In this work, two methods of analysis were developed for the determination of GUA, singly and in mixture, in spiked human plasma, and in several dosage forms. Such techniques are extremely green, sensitive, selective, easy to use, rapid, precise, and inexpensive. The first spectrofluorimetric method was utilized to measure GUA in human plasma, thanks to the high sensitivity achieved by this method and its dosage form. On the other hand, due to the significant overlap between these four components, spectrophotometric measurement is used in conjunction with a chemometric technique to determine the mixture of GUA, PARA, OXO, and SB simultaneously. Our methods are better in terms of greenness, as in this study we used spectroscopy, which has lower energy, lower waste, a safer solvent, and a shorter time than HPLC. Moreover, in our spectrofluorimetric method, no derivatization was needed, and 0.1 N HCl was used as a solvent, which is safer than methanol. Additionally, four highly overlapping drugs, “GUA, PARA, OXO, and SB” were determined using the spectrophotometric method rather than the HPLC method.

## Experimental

### Instrumentation

Shimadzu spectrofuorimeter RF-6000 (Kyoto, Japan), model R928 photomultiplier, 3D measurement function, with 1 cm quartz cuvettes, controlled from a PC using the Lab Solutions RF software for Windows. UVPC spectroscopy software version 3.7 (Shimadzu) was used to process absorption spectra using a double-beam Shimadzu 1601 PC UV–Vis spectrophotometer. PLS-toolbox for MATLAB 7.0.1.24704 (R14) Software was used to run PLS-2 analysis.

### Materials and reagents

Guaifenesin (GUA), Paracetamol (PARA), Oxomemazine (OXO), and Sodium Benzoate (SB), standards were provided by Amoun Pharmaceutical Company (Obour city, Cairo, Egypt), with purity 99.90%. All reagents were of analytical grade. Methanol, acetonitrile, and water, (Fisher, Cornal Lab, Cairo, Egypt). And 0.1 N HCl, 0.1 N sodium hydroxide, ethyl acetate, ethanol, cetermide, cyclodextrin, triton, calexerine and sodium lauryl sulfate were obtained from (Sigma Gmbh, Germany). phosphoric acid, glacial acidic acid and boric acid were used for the preparation of the Britton-Robinson buffer (universal buffer), obtained from (Sigma Gmbh, Germany).

VACSERA (Giza, Egypt) provided the frozen human plasma.

### Dosage forms

Gufidrexn tablet is labeled to contain 400 mg of GUA as an active ingredient with lactose monohydrate, microcrystalline cellulose PH101, croscarmellose sodium, magnesium stearate, and povidone K 30 as excipients.

Rectoplexil suppository is marked as having 66.6 mg of GUA, 66.6 mg of PARA, 66.6 mg of SB, and 3.3 mg of OXO.

### Standard solutions

Stock solutions (100 µg/mL) of GUA, PARA, OXO, and SB were obtained by accurately weighting 0.01gm, transferred into a 100-mL volumetric flask, and the volume was completed with methanol and stored at 4 °C.

### Procedure

#### Characterization of emission and excitation

For the spectrofluorimetric technique, a 1 µg/mL working solution was first scanned to obtain 3D spectra. Then, the excitation and emission wavelengths of GUA were recorded. A drug was scanned at those two wavelengths (λem = 302 nm following excitation at λex = 223 nm).

### Method validation

According to ICH criteria, the approach was validated^46^.

#### Linearity

For the spectrofluorimetric technique, aliquots from GUA working solution equivalent to (0.1–1.1 µg/mL) were accurately measured, transferred into a 10 mL volumetric flask, and completed to the final volume with 0.1N HCl (pH = 1.5). The fluorescence intensities at λem = 302 nm following excitation at λex = 223 nm were measured against blank. The fluorescence intensity values were computed and then plotted against their corresponding concentrations, and the regression equation was then determined. In all cases, the blank spectra were drawn and subtracted by the software from the corresponding spectra.

For the chemometric method, in four separate flasks, stock standard solutions were further diluted with methanol to obtain working solutions, each having a concentration of 10 µg/mL. The zero-order absorption spectrum of each sample was obtained against methanol as a blank in the UV range of 200–400 nm. The linearity of each component was checked using the normalized spectra. Twenty-five laboratory-prepared mixtures were prepared according to the five-level experimental design, containing different concentrations of the four cited compounds. These mixtures were prepared by mixing various aliquots from their standard solutions in sets of 10-mL volumetric flasks, and their volumes were completed to the mark using methanol to reach concentration ranges of (2.0–10.0) μg/mL for OXO, (4.0–16.0) μg/mL for GUA, (2.0–10.0) μg/mL for PARA, and (3.0–10.0) μg/mL for SB.

#### Accuracy

The accuracy of the developed spectrofluorimetric method was computed by calculating the % recovery ± SD of GUA (0.2, 0.3, 0.6, 0.7, and 0.9 μg/mL) under the previously mentioned procedures using the regression equation.

#### Precision

##### Repeatability (intraday precision)

Three concentrations of GUA (1.0, 0.6, and 0.2 µg/mL) were examined three times within the same day, using the previously mentioned procedures. The mean recovery% and %RSD were calculated.

##### Intermediate precision (interday precision)

The same three concentrations of GUA mentioned in repeatability were examined three times on three successive days, using the previously mentioned procedures. The mean recovery% and %RSD were calculated.

#### LOQ and LOD

For spectrofluorimetric method, LOD and LOQ were determined by means of analytical curve, SD of the intercept and the slope n expressed through following equation according to ICH guidelines^46^.

$$ {\text{LOD}}\, = \,{3}.{3}\sigma /{\text{S}}. $$

$$ {\text{LOQ}}\, = \,{1}0\sigma /{\text{S}}. $$

Here σ is the standard deviation of the intercept and S is the slope of the calibration curve.

### Bio-analytical validation

The spectrofluorimetric method was validated according to US Food and Drug Administration guidance for bio-analytical method validation^47^.

#### Accuracy and precision

It is crucial to assess precision and accuracy to make sure that our method is ready for validation. Our approach was used to measure accuracy and precision at four different QC levels: LLOQ, LOQ, MQC, and HQC, with concentrations of 0.1, 0.33, 0.55, and 0.77 µg/mL; respectively, for GUA on the same day for intraday accuracy and three successive days for inter-day accuracy. Every QC was assessed five times, after which the average was calculated. The accuracy was reported as mean recovery% ± SD and the precision as %RSD.

#### Stability

Stability was assessed at two QC levels, LQC and HQC, with concentrations of 0.3 and 0.77 µg/mL, respectively, for GUA. After determining each sample three times, the average recovery was computed.

For bench-top stability, it was evaluated by leaving spiked plasma samples at room temperature for 8 h. For freeze–thaw stability, plasma was frozen for 12 h, then thawed and used for the preparation of QCs. This procedure was applied at least three times. Finally, for auto- sampler stability, spiked plasma samples were left in the cuvette for 24 h.

#### Robustness

The ability of a method to remain stable even with small, intentional adjustments to its parameters is measured by its robustness. When the wavelength was changed by (± 0.1 nm), the evaluation was performed by computing the % RSD.

#### Matrix effect

It was applied to describe the reaction between samples and components found in the matrix. It was done at three levels of QCs (LQC, MQC, and HQC) with concentrations of 0.3, 0.55, and 0.77 µg/mL; respectively, for GUA. The fluorescence intensity of spiked samples was compared with that of pure samples and represented as %ME. When the %ME is greater than 100%, matrix components can have an enhancement effect; when the %ME is less than 100%, they can have a suppression effect.

#### Assay of dosage form

In the spectrofluorimetric method, gufidrexn tablets equivalent to 10 mg of GUA were weighed, quantitatively transferred to a 100 mL volumetric flask, dissolved in 60 mL of 0.1N HCl, sonicated for 30 min, and completed to the final volume with the same solvent. The resulting solution was filtered, and 0.1 N HCl (pH = 1.5) was used to create various dilutions. The emission spectra of the resulting dilutions were measured at 302 nm with excitation at 223 nm. The blank signal was then corrected. Also, standard addition was applied to ensure the validity of our method.

For Chemometric, one suppository was quantitatively transferred to a beaker, dissolved in 10.0 mL of methanol, and the mixture was left for 10.0 min in a water bath. The solution was filtrated after cooling through filter paper that had previously been wetted with methanol. 5 mL of methanol was added to the beaker that contains the solid fatty acid residue. This procedure was carried out three times, one milliliter of the previously prepared solution was added to a 250-mL volumetric, then completed to the final volume with methanol. The final concentrations were 10.0 µg/mL for GUA, PARA, and SB, and 0.5 µg/mL for OXO. 1.5 µg/mL from the standard of OXO was added to reach a concentration of 2 µg/mL. The concentrations were predicted by the developed model. Also, the standard addition method was applied to ensure the validity of our method.

#### Assay of spiked human plasma

For the Spectrofluorimetric method, in centrifuge tubes, different concentrations were spiked into 1 mL of human plasma, followed by the addition of 4 mL of acetonitrile, vortexing for 60 s, and centrifugation at 6,000 rpm for 15 min. The supernatant was then removed, and RFI was measured at λ emission 302 nm using λ excitation 223 nm. Blank plasma was done simultaneously.

## Results and discussion

**Figure 2:**
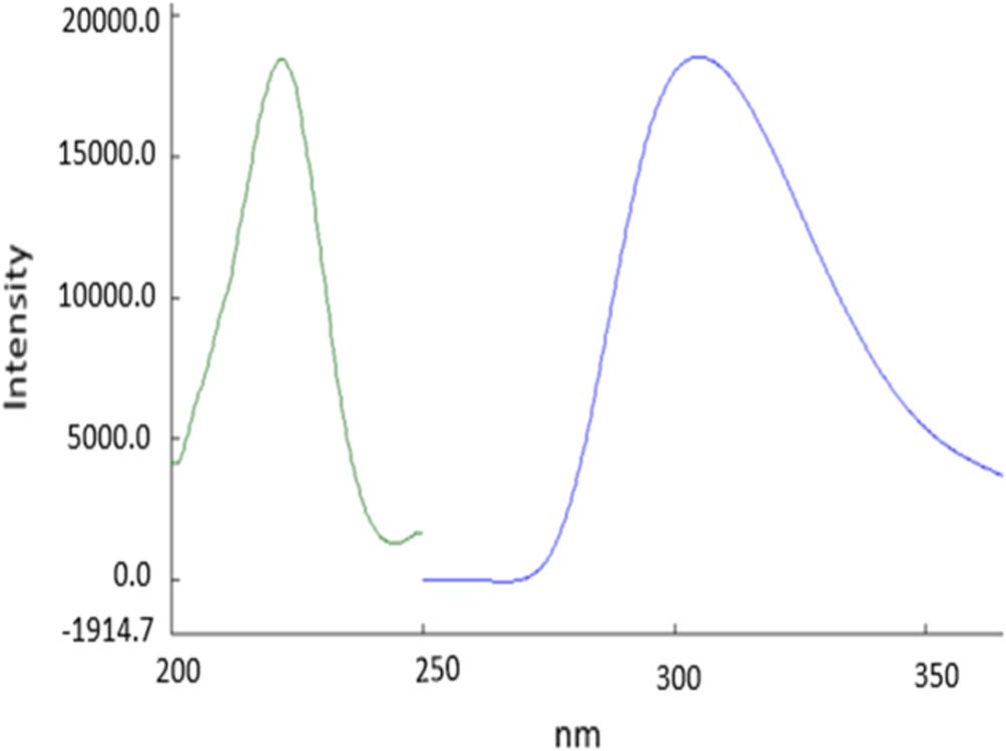
Excitation and emission spectra of GUA in 0.1**N** HCl at (λex 223) and (λem 302), respectively using the proposed spectrofluorometric method at 1 μg/mL.

*Spectrofluorimetric method*, as shown in (Fig. 2), GUA exhibits strong natural fluorescence in 0.1 N HCl at emission 302 nm following excitation at λex = 223 nm. The effects of several experimental variables on the fluorescence intensities of the medicine under study were examined, and the ideal settings were chosen to get the highest intensity.

### Effect of solvents on the fluorescence intensity of GUA

**Figure 3:**
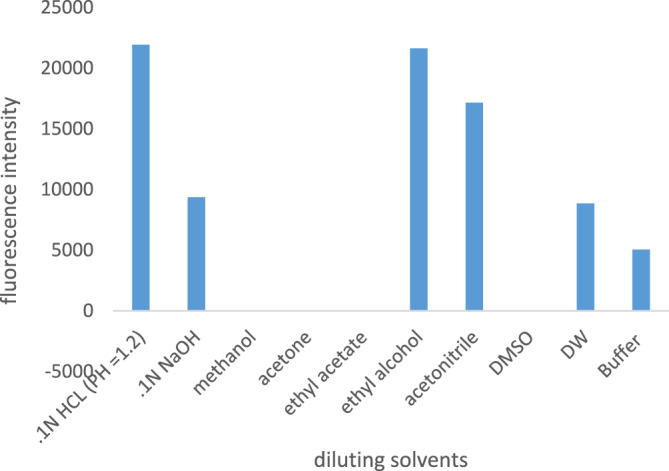
The effect of different solvents in the fluorescence intensity of guaifenesin.

Several solvents were investigated, such as water, methanol, ethanol, ethyl acetate, acetone, acetonitrile, universal buffer, 0.1 N HCl, and 0.1 N NaOH. The results, shown in (Fig. 3) revealed that the highest intensity was obtained by using 0.1N HCl.

### Effect of pH on the fluorescence intensity of GUA

**Figure 4:**
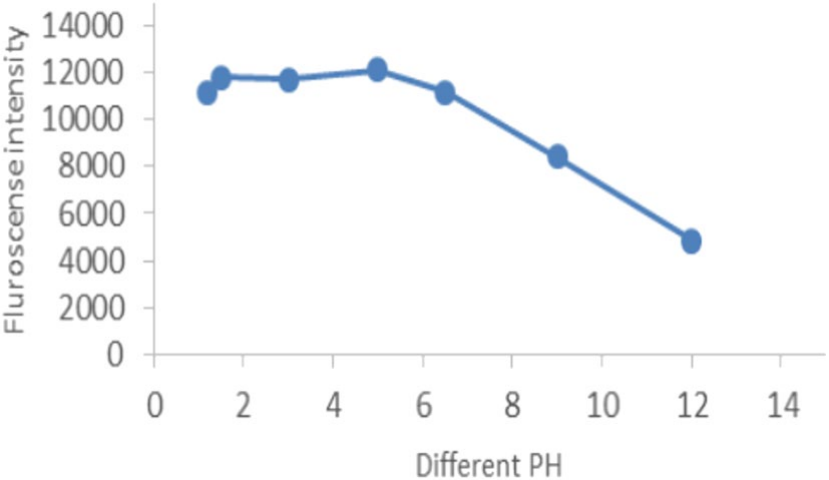
Effect of different PH in the fluorescence intensity of guaifenesin.

Different pH ranges (1.5 to 12) were studied by adding BR universal buffer and 0.1 N NaOH. As shown in (Fig. 4), the highest intensity was obtained in the pH range (1.5–5), and increasing the pH will decrease the fluorescence intensity.

**Figure 5:**
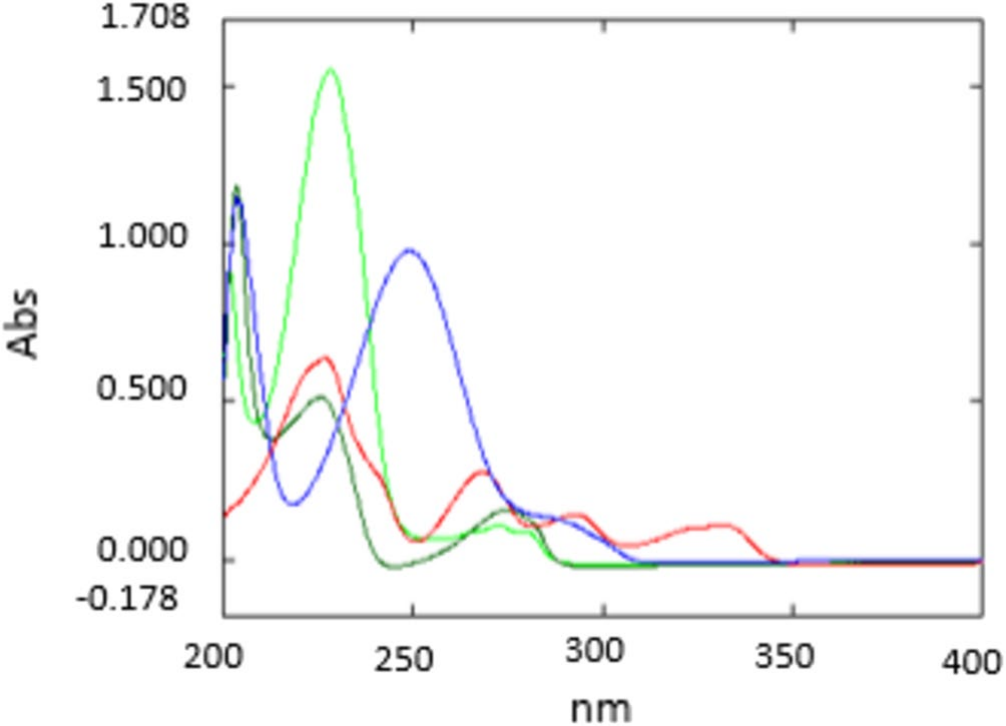
Absorption spectra of (a) OXO, (b) GUA, (c) PARA, and (d) SB at wavelength range from 200 to 400 nm with concentration 10 µg/mL.

*Chemometric method*, due to the severe overlap between the spectra of the four studied components, (Fig. 5). Multivariant spectrophotometry is the technique of choice to resolve this overlap. It was found that readings below 230.0 nm showed high noise, which may affect the obtained result, otherwise, readings above 300.0 nm gave almost zero absorption, leading to invaluable information, so the range between 230.0 nm to 300.0 nm was the range of choice in the calculation. As per the multilevel multifactor design (five level calibration design) outlined by Brereton, mixtures having various aliquots of the four specified components were created^48^.

At 0.2 nm intervals, the acquired spectra of the prepared solutions were scanned from 200.0 to 400.0 nm. Utilizing MATLAB, the data were examined, where multivariate calibration models were built by using seventeen mixtures from the previously prepared solutions to build the model and eight laboratory-prepared mixtures were utilized as a set for external validation (specificity), as shown in (Table 1). To create the PLs, all the calibration spectral data were mean-centered, and the cross-validation technique "leave-one out" was applied. For MCR-ALS, applied constraints were the critical optimization parameter; a non-negativity constraint (non-negativity least-squares) was applied to the concentration profile with an equality constraint (equal than) to obtain satisfactory parameters.

**Table 1:** Calibration and validation set in the PLS-2 method.

Mixture number	GUA	PARA	SB	OXO
1ª	10.00	6.00	6.50	10.00
2	10.00	2.00	3.00	10.00
3	4.00	2.00	10.00	4.00
4	4.00	10.00	4.75	10.00
5	16.00	4.00	10.00	6.00
6ª	7.00	10.00	6.50	4.00
7ª	16.00	6.00	4.75	4.00
8ª	10.00	4.00	4.75	8.00
9	7.00	4.00	8.25	10.00
10	7.00	8.00	10.00	8.00
11ª	13.00	10.00	8.25	6.00
12ª	16.00	8.00	6.50	10.00
13ª	13.00	6.00	10.00	10.00
14	10.00	10.00	10.00	2.00
15	16.00	10.00	3.00	8.00
16	16.00	2.00	8.25	2.00
17	4.00	8.00	3.00	6.00
18	13.00	2.00	6.50	8.00
19	4.00	6.00	8.25	8.00
20ª	10.00	8.00	8.25	4.00
21	13.00	8.00	4.75	2.00
22	13.00	4.00	3.00	4.00
23	7.00	2.00	4.75	6.00
24	4.00	4.00	6.50	2.00
25	7.00	6.00	3.00	2.00

ªvalidation set.

### Partial least squares (PLS) model

**Figure 6:**
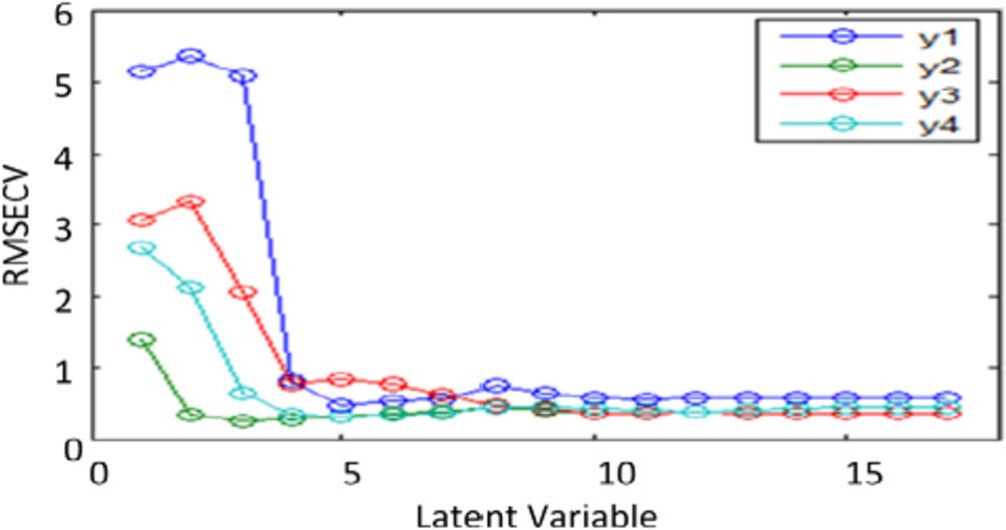
RMSECV plot of the cross-validation results of the training set as a function of the number of principle components used to construct the PLS-2 calibration using zero order spectra of y1(GUA), y2(PARA), y3(SB) and y4(OXO).

**Figure 7:**
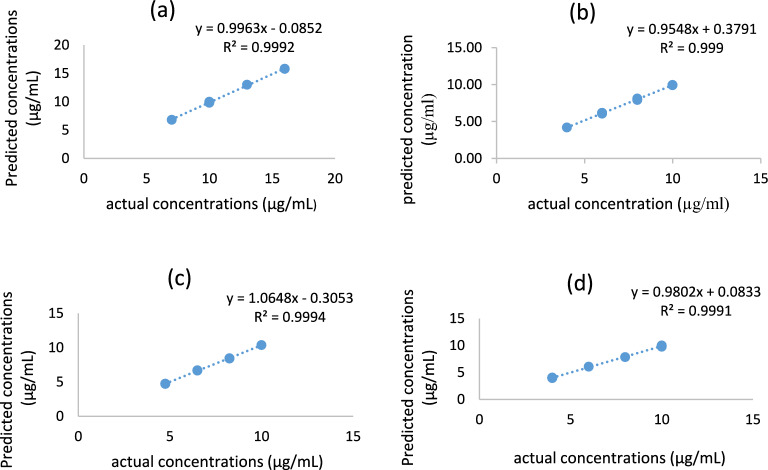
Predicted versus actual concentrations (μg/mL) plot for (**a**) GUA, (**b**) PARA, (**c**) SB and (**d**) OXO in the validation set using the PLS-2 method. Y = predicted concentration μg/mL. X = actual concentration μg/mL.

**Figure 8:**
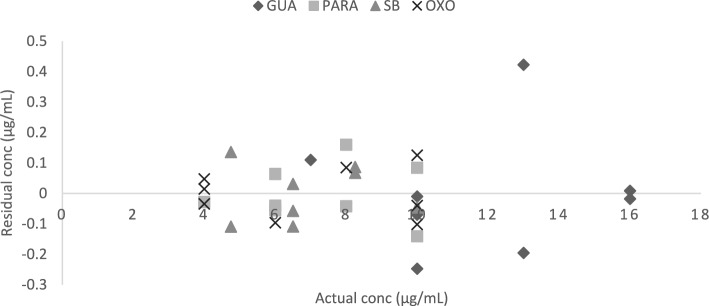
Residual versus actual concentrations (μg/mL) plot for GUA, PARA, SB and OXO in the validation set using the PLS-2 method.

For PLS, 5 factors were found to be the absolute number of latent variables to explain by the built model, as illustrated in (Fig. 6)^49^. By showing known versus predicted concentrations for each chemical in the constructed model, the predictive power of the model was evaluated. The correlation coefficients of 0.9992, 0.999, 0.9994, and 0.9991 found for GUA, PARA, SB, and OXO, respectively, show that each chemical showed a linear connection (Fig. 7). Also, the created model was applied to an external validation set for the determination of the four components to assess its predictive power (specificity). The created actual concentrations and residual concentrations were displayed (Fig. 8), and it appeared that the residuals were randomly distributed around zero for all samples. The values of the root mean square error of prediction (RMSEP), the recoveries, and the mean recoveries are displayed in (Table 2). Statistical parameters using the improved PLS-2 method are shown in (Table 3).

**Table 2:** Percent recoveries and RMSEP of GUA, PARA, SB, and OXO in the validation set using the PLS-2 method.

Mixture number	GUA	PARA	SB	OXO
1	100.51	100.78	103.73	99.87
6	95.95	101.69	104.55	98.87
7	98.49	102.48	104.78	100.88
8	98.16	104.42	99.42	98.03
11	95.12	99.48	101.96	100.96
12	98.65	101.39	102.31	99.27
13	99.84	102.73	103.70	97.64
20	98.75	98.92	102.20	99.68
Rec%	98.18	100.78	102.83	99.40
RMSEP^a^	0.28	0.13	0.23	0.11

^a^Root mean square error of prediction.

**Table 3:** Statistical parameters for simultaneous determination of GUA, PARA, SB, and OXO using the optimized PLS-2 method with zero order spectra.

Parameter	PLS-2 method with zero order			
	GUA	PARA	SB	OXO
Conc range (µg/ml)	4.0–16.0	2.0–10.0	3.0–10.0	2.0–10.0
No of factors	5	5	5	5
SEP^a^	0.33	0.15	0.26	0.12
RMSEP^b^	0.28	0.13	0.23	0.11
Intercept^c^	− 0.0852	0.3791	− 0.3053	0.0833
Slope^c^	0.9963	0.9548	1.0648	0.9802
r square^c^	0.9992	0.9990	0.9994	0.9991

^a^Square error of prediction.

^b^Root mean square error of prediction.

^c^Data of the straight line plotted between predicted concentrations of each component versus actual concentrations.

### Multivariate curve resolution (MCR) alternating least-squares (ALS) model

MCR is a technique evolved from factor analysis that is predicated on a bilinear model^50^. In MCR, the measured spectra data matrix is decomposed into the concentration and spectra-profile matrixes of the pure components in the samples, and then error is calculated. Repetitive estimations of concentrations from the spectra profile and vice versa. MCR were optimized by the ALS procedure. Because data matrix decomposition has no unique solution, the number of possible solutions could be minimized by applying constraints like unimodality, closure, equality, or non-negativity. In this work, a non-negativity constraint was applied to the concentration profile, which necessitated the concentration to be equal to or greater than zero. Also, non-negativity was applied to the spectra constraint. The generated MCR-ALS models were tested using the validation set spectrum to gauge their level of predictability. (Table 4) displays the calculated mean recoveries and RSDs for each component. (Table 5) displays the regression parameters, standard error of prediction (SEP), and root mean square error of prediction (RMSEP) for the validation sets. Near values of RMSEP and SEP showed that the suggested calibration models did not exhibit any overfitting.

**Table 4:** Percent recoveries and RMSEP of GUA, PARA, SB, and OXO in the validation set using the MCR-ALS method.

Mixture number	GUA	PARA	SB	OXO
1	100.83	99.00	97.13	101.00
6	100.00	98.00	100.00	99.75
7	100.63	101.67	98.53	102.83
8	100.00	99.75	98.95	98.75
11	99.23	101.55	96.85	99.83
12	100.00	101.25	100.00	99.90
13	99.92	101.67	101.00	101.00
20	99.00	100.42	98.18	103.31
Rec%	99.95	100.41	98.83	100.94
RMSEP^a^	0.07	0.11	0.13	0.08

^a^Root mean square error of prediction.

**Table 5:** Statistical parameters for simultaneous determination of GUA, PARA, SB, and OXO using the optimized MCR-ALS method with zero order spectra.

Parameter	MCR-ALS method with zero order			
	GUA	PARA	SB	OXO
Conc. range (µg/ml)	4.0–16.0	2.0–10.0	3.0–10.0	2.0–10.0
SEP^a^	0.07	0.11	0.13	0.08
RMSEP^b^	0.07	0.11	0.13	0.08
Intercept^c^	− 0.0561	− 0.0866	− 0.0426	− 0.0492
Slope^c^	1	1	1	1
r Square^c^	0.9997	0.9998	0.9995	0.9998

^a^Square error of prediction.

^b^Root mean square error of prediction.

^c^Data of the straight line plotted between predicted concentrations of each component versus actual concentrations.

**Figure 9:**
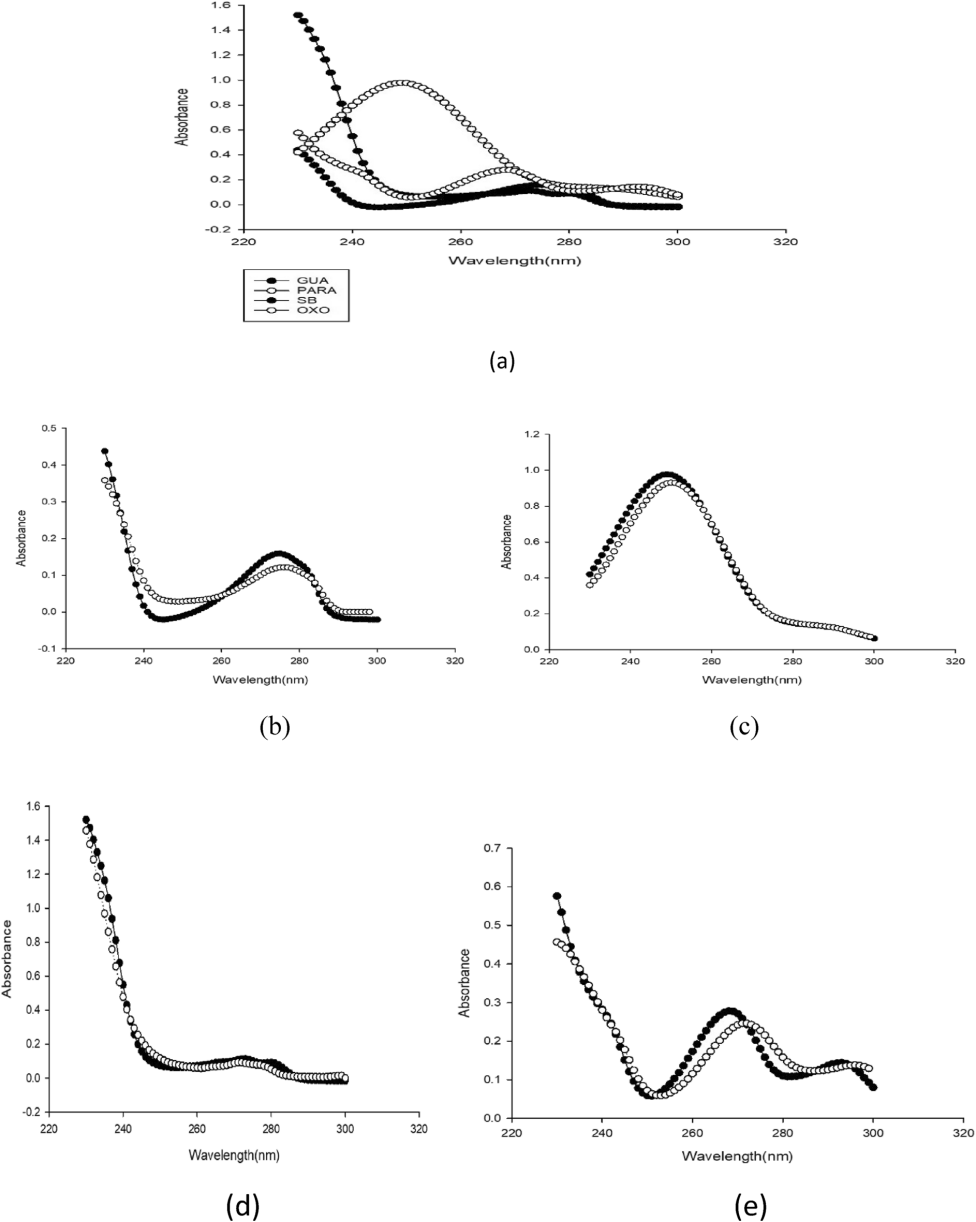
(**a**) zero order spectrum of four determined drugs at wavelength range from 230 to 300 nm. The zero order spectra (bold dotted line) and extracted spectra (dotted line) by MCR-ALS for (**b**) GUA, (**c**) PARA, (**d**) SB, and (**e**) OXO.

The MCR-ALS models provided a qualitative meaning in their algorithms; therefore, the spectral profiles of drugs could be estimated. The spectra obtained from the model were compared with pure spectra (Fig. 9).

### Method validation

#### Linearity

The relative fluorescence intensity (RFI) and its corresponding concentrations were found to be linearly correlated, and the regression equation's parameters were then determined. As shown in (Table 6).

**Table 6:** Assay parameters and method validation for the determination of pure sample of GUA by the proposed spectrofluorometric method.

Parameter	Value
Range µg/ml	0.1–1.1
Regression equation (Y = bCi + a)^a^ Correlation coefficient	y = 21537c + 337.2 0.9993
Slope	21,537
Intercept	337.2
LOD µg/ml	0.03
LOQ µg/ml	0.08
Accuracy (mean recovery % ± SD)	100.64 ± 1.55
Repeatability (%RSD)	0.27
Intermediate precision (%RSD)	1.85

^a^*a* Intercept, *b* slope, and *Ci* concentration of drug in μg/mL, y (RFI).

#### Accuracy

The results, which are displayed in (Table 6) including the mean recovery and standard deviation, demonstrated the accuracy of the suggested technique.

#### Precision

Due to the low values of RSD%, which were calculated for the three concentrations of GUA and found to be less than 2.0% in all three concentrations, as shown in (Table 6), the suggested method's precision is acceptable.

#### LOQ and LOD

LOD and LOQ were found using the suggested method in accordance with ICH guidelines^46^, as shown in (Table 6).

### Bio-analytical validation

#### Accuracy and precision

The results displayed in (Tables 7, 8) showed low SD and low % RSD, which demonstrated the accuracy and precision of the suggested method and enabled us to determine the proposed analyte in the human plasma.

**Table 7:** Percent recoveries and standard division results of accuracy in human plasma.

Samples	Conc. (µg/ml)	% Recovery
LLOQ^a^	0.1	100.05
LQC^b^	0.3	100.46
MQC^c^	0.55	101.83
HQC^d^	0.77	101.69
%Mean ± SD	101.01 ± 0.89	

^a^Lower limit of quantification, ^b^Low QC, ^c^Medium QC, ^d^High QC.

**Table 8:** Results of precision in human plasma.

	Samples	Conc. (µg/ml )	Mean %recovery ± RSD
Intraday	LLOQ^a^	0.1	100.07 ± 0.02
	LQC^b^	0.3	99.95 ± 0.89
	MQC^c^	0.55	101.55 ± 0.48
	HQC^d^	0.77	101.70 ± 0.01
Inter-day	LLOQ^a^	0.1	100.21 ± 0.12
	LQC^b^	0.3	101.42 ± 1.27
	MQC^c^	0.55	101.85 ± 1.88
	HQC^d^	0.77	101.43 ± 0.50

^a^Lower limit of quantification, ^b^Low QC, ^c^Medium QC, ^d^High QC.

#### Stability

GUA stability in human plasma was evaluated using bench-top, freeze–thaw, and auto-sampler stability. The results obtained showed good selectivity (Table 9).

**Table 9:** Stability results of GUA in human plasma under different stability assessment conditions.

	Samples	Conc. (µg/ml )	Mean × %recovery ± RSD
Bench-top	LQC^a^	0.3	100.60 ± 0.13
	HQC^b^	0.77	101.70 ± 0.60
Freeze–thaw	LQC^a^	0.3	99.74 ± 1.13
	HQC^b^	0.77	101.57 ± 0.89
Auto-sampler	LQC^a^	0.3	99.18 ± 1.66
	HQC^b^	0.77	100.02 ± 1.87

^a^Low QC, ^b^High QC.

#### Robustness

Low %RSD was established after a small change in the method wavelength (Table 10).

**Table 10:** Robustness results of GUA in human plasma.

	Samples	Conc. (µg/ml)	%Recovery
Wavelength change (± 0.1 nm)	LQC^a^	0.3	99.18
	HQC^b^	0.77	100.02
	%recovery ± RSD	99.60 ± 0.59	

^a^Low QC, ^b^High QC.

#### Matrix effect

% ME was found to be 102.32 for GUA. The results obtained mean that components in the matrix cause enhancement effects.

#### Assay of dosage form

For the Spectrofluorimetric method, to determine the nominal contents, the corresponding regression equation was employed, and the standard addition procedure was used. The results are shown in (Table 11).

**Table 11:** Determination of GUA in gufidrexn® tablets 400 mg and application of standard addition technique.

Claimed conc. µg/ml	Found conc. µg/ml	Recovery%	Standard addition		
			Added µg/ml	Found µg/ml	Recovery%
1.00	1.00	100.44	0.20	0.20	102.35
0.70	0.70	100.40	0.30	0.30	101.48
0.30	0.30	100.48	0.40	0.40	100.16
Mean ± SD		100.44 ± 0.04			101.33 ± 1.10

For Chemometric PLS and MCR-ALS, the mean recoveries and RSD% of GUA, PARA, SB, and OXO in rectoplexil supp were calculated using the prior models, as shown in (Tables 12, 13).

**Table 12:** Assay results of GUA, PARA, SB, and OXO in its commercial preparations using the PLS-2 method.

PLS-2 method				
Component	Recovery % ± SD	Standard addition technique		
		Added µg/ml	Found µg/ml	Recovery %
GUA	98.49 ± 0.50	11.00	10.91	99.20
		13.00	13.04	100.29
		15.00	15.00	99.99
Mean ± SD				99.83 ± 0.57
PARA	98.51 ± 0.64	2.00	2.00	99.80
		4.00	3.97	99.20
		6.00	6.07	101.13
Mean ± SD				100.04 ± 0.99
SB	98.13 ± 0.51	3.00	2.90	96.51
		6.00	5.91	98.47
		8.00	7.95	99.39
Mean ± SD				98.12 ± 1.47
OXO	100.21 ± 0.36	4.00	4.04	100.88
		6.00	6.07	101.19
		8.00	7.98	99.81
Mean ± SD				100.63 ± 0.72

**Table 13:** Assay results of GUA, PARA, SB, and OXO in its commercial preparations using the MCR-ALS method.

MCR-ALS method				
Component	Recovery % ± SD	Standard addition technique		
		Added µg/ml	Found µg/ml	Recovery %
GUA	99.19 ± 0.2	11.00	11.1	100.91
		13.00	13.2	101.54
		15.00	15.2	101.33
Mean ± SD				101.26 ± 0.32
PARA	100.24 ± 0.12	2.00	1.99	99.50
		4.00	3.99	99.75
		6.00	6	100.00
Mean ± SD				99.75 ± 0.25
SB	98.61 ± 0.32	3.00	3.00	100.00
		6.00	5.99	99.83
		8.00	8	100.00
Mean ± SD				99.99 ± 0.10
OXO	101.39 ± 0.45	4.00	4.00	100.00
		6.00	5.96	99.33
		8.00	7.99	99.88
Mean ± SD				99.74 ± 0.35

#### Assay of drug in human plasma

The spectrofluorimetric method was utilized to successfully determine the presence of GUA in human plasma successfully with accepted %recovery and SD, as shown in (Table 14).

**Table 14:** Determination of GUA in spiked human plasma.

Spiked conc. µg/ml	Found conc. µg/ml	Recovery %
0.20	0.20	100.33
1.08	1.10	101.83
1.10	1.11	100.93
Mean ± SD		101.03 ± 0.75

### Evaluation of the methods greenness

The methods' level of greenness was assessed using the AGREE calculator and the analytical Eco-scale. Van-Aken et al.^51^ presented the Eco-Scale, a highly intriguing method for evaluating green organic synthesis. They assumed that the "ideal" reaction would have a score of 100 on the Eco-Scale. Eco-scale starts subtracting points from the perfect score of 100 for the use of chemicals, instruments, energy, and waste produced.

The primary benefit of Eco-scale is that it is safe for the environment and the operator to use, uses low-cost compounds, and can be done at room temperature with a 100% yield. The overall score is decreased by the penalty points given for any parameter that deviates from "the ideal value." The eco-friendlier and more cost-effective, the higher the score. This concept can be adapted to evaluate green analytical methods^52^. The drawbacks of Eco-scale, experience and intuition are frequently the only sources of information used rather than "exact science" to determine the weight of each characteristic and, the relative worth of the penalty points^53^.

The 12 Principles of Green Analytical Chemistry are referenced in the AGREE calculator's input criteria, which helps to overcome these restrictions. Every one of the twelve input variables is converted into a standard scale with a range of 0–1^54^. The straightforward red-yellow-green color scale represents each principle's method performance, and the breadth of the section that corresponds to each principle represents its weight. The entire score is displayed in the center of the pictogram, with values nearing 1 and a dark green tint signifying that the evaluated process is more environmentally friendly^55^.

The analytical Eco-scale score and penalty points received a score of 86 for two approaches, which is higher than the cutoff of 75. These analytical approaches can be categorized as exceptional green ways of analysis according to this metric system (Table 15).

**Table 15:** Results of Eco-scale assessment for chemometric method and spectrofluorometric method.

Hazard	Penalty points for chemometric method	Penalty points for spectrofluorometric method
Reagents: methanol 0.1 N HCl	6	6
Instruments: energy and occupational hazard	0	0
Waste	8	8
Total penalty points	14	14
Analytical eco-scale score	86	86

**Figure 10:**
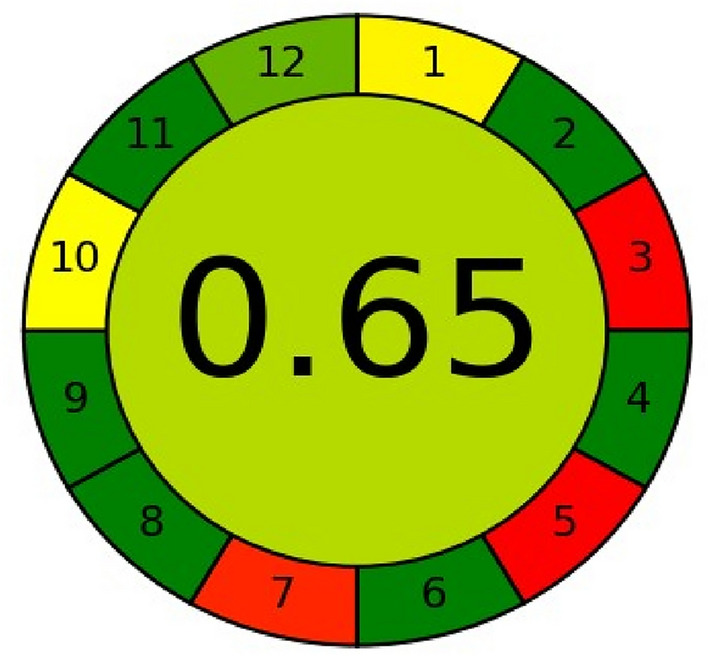
Results of AGREE assessment for chemometric method.

**Figure 11:**
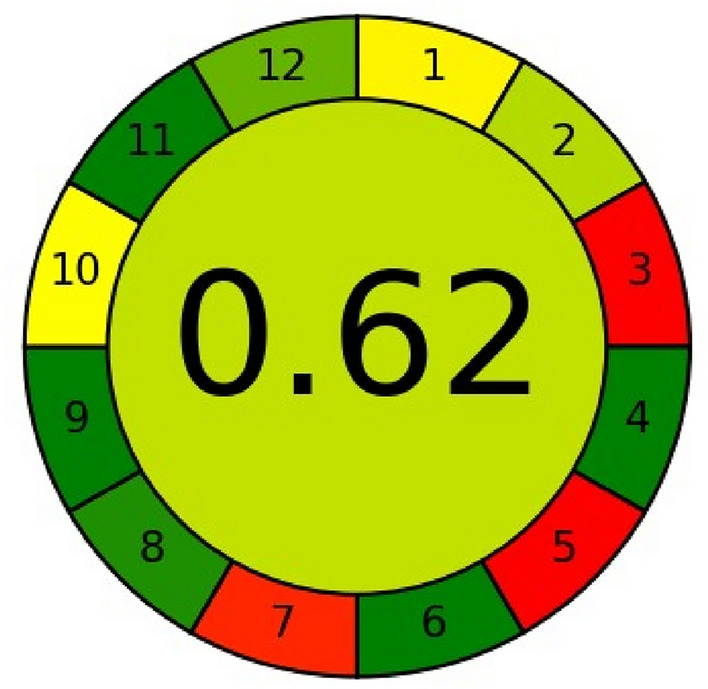
Results of AGREE assessment for spectrofluorometric method.

Our proposed approaches may be classified as green methods because they have overall scores of 0.65 and 0.62 for the chemometric method and the spectrofluorometric method, respectively. Both approaches have a middle color of green. It's also evident that while principles 3.5 and 7 are colored red, the majority of the 12 principles are green. This is because the suggested approach produces waste, is not automated or miniaturized, and operates offline (Figs. 10, 11).

### Comparison of chemometric approach with old reported chromatographic method

Comparing our study with previous study^19^ in terms of greenness using the Agree Calculator. The two works have the same application. This study is better in terms of greenness, as we used UV–Vis spectroscopy, which has lower energy, lower waste, a safer solvent, and a shorter time than HPLC. The previously reported method score was 0.53, but in our study, the score was 0.65. These results indicate that our study is greener than the previously reported study (Table 16).

**Table 16:** Results of AGREE assessment for reported methods and proposed methods.

Method	AGREE
Reported HPLC-DAD (14) Stationary phase: C18 Mobile phase: acetonitrile: methanol: phosphate buffer (20:5:75 v/v) Flow rate: 1.2 mL/min Run time: 12.0 min	
Reported spectrofluorometric method (41) Solvent: methanol Time : 3 Min Derivatization: Yes	
Proposed spectrofluorimetric method Solvent: 0.1 N HCL Time: 3 Min Derivatization: NO	
Proposed chemometric method Solvent: methanol Time: 3 Min	

### Comparison of spectrofluorimetric approach with old reported spectrofluorimetric method

Comparing our study with previously reported study^43^ using the Agree Calculator. The previously reported method score was 0.59, but in our study, the score was 0.62. This study is greener, as in our study we did not use any derivatization method, but in the reported study derivatization method was used. Also, in our study, we used 0.1 N HCl as a solvent, which is safer than the methanol that was used in a previously reported study (Table 16).

## Conclusion

A green, straightforward, accurate, affordable, quick, and sensitive spectrofluorimetric approach was created to detect GUA in spiked human plasma and tablet dosage form. A spectrophotometric technique with chemometric assistance was developed for the simultaneous detection of four significant overlapped drugs (GUA, PARA, SB, and OXO) in their lab mixtures and suppository dosage forms. Two models were applied, PLS and MCR-ALS, for solving the severe overlap of our components. MCR-ALS allowed us to perform quantitative and qualitative analysis of our components with good recovery when compared with the PLS model. The optimal conditions for high fluorescence intensity were dissolving the material in 0.1 N HCl (pH = 1.5). Moreover, raising the pH eliminated the intensity. In comparison to the described HPLC approach, the two methods are thought to be more efficient, affordable, and greener. Using spectroscopic techniques makes our study better in terms of greenness. Moreover, in the spectrofluorimetric approach no derivatization was required, and 0.1 N HCl was used as a solvent which is safer than methanol.

## Data Availability

The datasets used and/or analysed during the current study available from the corresponding author (Name: Marwa Hassan Mohamed, Gmail: marwahasan.mohamed@pharma.asu.edu.eg) on reasonable request.
